# Supplementary material for: HIVprotI: an integrated web based platform for prediction and design of HIV proteins inhibitors
Source: J Cheminform. 2018 Mar 9;10:12. doi: 10.1186/s13321-018-0266-y (PMC5845081; doi:10.1186/s13321-018-0266-y)
Supplement: Supplementary file 2 — Additional file 2. Source code of HIVProtI web server. [file 13321_2018_266_MOESM2_ESM.zip › HIVprotI_Source-code/hivprotI/weka-3-6-6/doc/weka/datagenerators/clusterers/SubspaceClusterDefinition.html]

SubspaceClusterDefinition


---


|  |  |  |
| --- | --- | --- |
| **Overview** \| **Package** \| **Class** \| **Tree** \| **Deprecated** \| **Index** \| **Help** \| **Weka's home** | |  |
| **PREV CLASS**   NEXT CLASS | **FRAMES**    **NO FRAMES**     **All Classes** |  |
| SUMMARY: NESTED \| FIELD \| CONSTR \| METHOD | DETAIL: FIELD \| CONSTR \| METHOD |  |


---


## weka.datagenerators.clusterers Class SubspaceClusterDefinition

```
java.lang.Object
  weka.datagenerators.ClusterDefinition
      weka.datagenerators.clusterers.SubspaceClusterDefinition
```

**All Implemented Interfaces:**: java.io.Serializable, OptionHandler, RevisionHandler

---

``` public class SubspaceClusterDefinition extends ClusterDefinition ```

A single cluster for the SubspaceCluster datagenerator


Valid options are:

```
 -A <range>
  Generates randomly distributed instances in the cluster.
```

```
 -U <range>
  Generates uniformly distributed instances in the cluster.
```

```
 -G <range>
  Generates gaussian distributed instances in the cluster.
```

```
 -D <num>,<num>
  The attribute min/max (-A and -U) or mean/stddev (-G) for
  the cluster.
```

```
 -N <num>..<num>
  The range of number of instances per cluster (default 1..50).
```

```
 -I
  Uses integer instead of continuous values (default continuous).
```

**Version:**
:   $Revision: 1.5 $

**Author:**
:   Gabi Schmidberger (gabi@cs.waikato.ac.nz), FracPete (fracpete at waikato dot ac dot nz)

**See Also:**: `SubspaceCluster`, Serialized Form

---

**Constructor Summary**

`SubspaceClusterDefinition()`
  
          initializes the cluster, without a parent cluster (necessary for GOE)

`SubspaceClusterDefinition(ClusterGenerator parent)`
  
          initializes the cluster with default values


| **Method Summary** | |
| --- | --- |
| `java.lang.String` | `attributesToString()`             Make a string from the attribues list. |
| `java.lang.String` | `attrIndexRangeTipText()`             Returns the tip text for this property |
| `java.lang.String` | `clusterSubTypeTipText()`             Returns the tip text for this property |
| `java.lang.String` | `clusterTypeTipText()`             Returns the tip text for this property |
| `boolean[]` | `getAttributes()` |
| `java.lang.String` | `getAttrIndexRange()`             returns the attribute range(s). |
| `SelectedTag` | `getClusterSubType()`             Gets the cluster sub type. |
| `SelectedTag` | `getClusterType()`             Gets the cluster type. |
| `int` | `getMaxInstNum()`             Gets the upper boundary for instances per cluster. |
| `double[]` | `getMaxValue()` |
| `java.lang.String` | `getMeanStddev()`             returns the current mean/stddev setup |
| `double[]` | `getMeanValue()` |
| `int` | `getMinInstNum()`             Gets the lower boundary for instances per cluster. |
| `double[]` | `getMinValue()` |
| `int` | `getNumInstances()` |
| `java.lang.String[]` | `getOptions()`             Gets the current settings of the datagenerator BIRCHCluster. |
| `java.lang.String` | `getRevision()`             Returns the revision string. |
| `double[]` | `getStddevValue()` |
| `java.lang.String` | `getValuesList()`             returns the range for each attribute as string |
| `java.lang.String` | `globalInfo()`             Returns a string describing this data generator. |
| `boolean` | `isContinuous()`             checks, whether cluster sub type is continuous |
| `boolean` | `isGaussian()`             checks, whether cluster type is gaussian |
| `boolean` | `isInteger()`             checks, whether cluster sub type is integer |
| `boolean` | `isRandom()`             checks, whether cluster type is random |
| `boolean` | `isUniform()`             checks, whether cluster type is uniform |
| `java.util.Enumeration` | `listOptions()`             Returns an enumeration describing the available options. |
| `java.lang.String` | `maxInstNumTipText()`             Returns the tip text for this property |
| `java.lang.String` | `meanStddevTipText()`             Returns the tip text for this property |
| `java.lang.String` | `minInstNumTipText()`             Returns the tip text for this property |
| `void` | `setAttrIndexRange(java.lang.String rangeList)`             Sets which attributes are used in the cluster attributes among the selection will be discretized. |
| `void` | `setClusterSubType(SelectedTag value)`             Sets the cluster sub type. |
| `void` | `setClusterType(SelectedTag value)`             Sets the cluster type. |
| `void` | `setMaxInstNum(int newMaxInstNum)`             Sets the upper boundary for instances per cluster. |
| `void` | `setMeanStddev(java.lang.String meanstddev)`             Sets mean and standarddeviation. |
| `void` | `setMinInstNum(int newMinInstNum)`             Sets the lower boundary for instances per cluster. |
| `void` | `setNumInstances(java.util.Random r)`             Sets the real number of instances for this cluster. |
| `void` | `setOptions(java.lang.String[] options)`             Parses a list of options for this object. |
| `void` | `setParent(SubspaceCluster parent)`             sets the parent datagenerator this cluster belongs to |
| `void` | `setValuesList(java.lang.String fromToList)`             Sets the ranges for each attribute. |
| `void` | `setValuesList(java.lang.String fromToList, double[] first, double[] second, java.lang.String optionLetter)`             Sets the ranges for each attribute. |
| `java.lang.String` | `toString()`             Make a string from the cluster features. |
| `java.lang.String` | `valuesListTipText()`             Returns the tip text for this property |

**Methods inherited from class weka.datagenerators.ClusterDefinition**

`getParent, parentTipText, setParent`

**Methods inherited from class java.lang.Object**

`equals, getClass, hashCode, notify, notifyAll, wait, wait, wait`

**Constructor Detail**

### SubspaceClusterDefinition

```
public SubspaceClusterDefinition()
```

:   initializes the cluster, without a parent cluster (necessary for GOE)

---


### SubspaceClusterDefinition

```
public SubspaceClusterDefinition(ClusterGenerator parent)
```

:   initializes the cluster with default values

    **Parameters:**: `parent` - the datagenerator this cluster belongs to


**Method Detail**

### globalInfo

```
public java.lang.String globalInfo()
```

:   Returns a string describing this data generator.

    :   **Overrides:**: `globalInfo` in class `ClusterDefinition`
    :   **Returns:**: a description of the data generator suitable for displaying in the explorer/experimenter gui

---


### listOptions

```
public java.util.Enumeration listOptions()
```

:   Returns an enumeration describing the available options.

    :   **Specified by:**: `listOptions` in interface `OptionHandler` **Specified by:**: `listOptions` in class `ClusterDefinition`
    :   **Returns:**: an enumeration of all the available options

---


### setOptions

```
public void setOptions(java.lang.String[] options)
                throws java.lang.Exception
```

:   Parses a list of options for this object.


    Valid options are:

    ```
     -A <range>
      Generates randomly distributed instances in the cluster.
    ```

    ```
     -U <range>
      Generates uniformly distributed instances in the cluster.
    ```

    ```
     -G <range>
      Generates gaussian distributed instances in the cluster.
    ```

    ```
     -D <num>,<num>
      The attribute min/max (-A and -U) or mean/stddev (-G) for
      the cluster.
    ```

    ```
     -N <num>..<num>
      The range of number of instances per cluster (default 1..50).
    ```

    ```
     -I
      Uses integer instead of continuous values (default continuous).
    ```

    :   **Specified by:**: `setOptions` in interface `OptionHandler` **Specified by:**: `setOptions` in class `ClusterDefinition`
    :   **Parameters:**: `options` - the list of options as an array of strings **Throws:**: `java.lang.Exception` - if an option is not supported

---


### getOptions

```
public java.lang.String[] getOptions()
```

:   Gets the current settings of the datagenerator BIRCHCluster.

    :   **Specified by:**: `getOptions` in interface `OptionHandler` **Specified by:**: `getOptions` in class `ClusterDefinition`
    :   **Returns:**: an array of strings suitable for passing to setOptions

---


### attributesToString

```
public java.lang.String attributesToString()
```

:   Make a string from the attribues list.

    :   **Returns:**: the attributes as string

---


### toString

```
public java.lang.String toString()
```

:   Make a string from the cluster features.

    :   **Overrides:**: `toString` in class `ClusterDefinition`
    :   **Returns:**: the cluster features as string

---


### setParent

```
public void setParent(SubspaceCluster parent)
```

:   sets the parent datagenerator this cluster belongs to

    :   **Parameters:**: `parent` - the parent datagenerator

---


### setAttrIndexRange

```
public void setAttrIndexRange(java.lang.String rangeList)
```

:   Sets which attributes are used in the cluster
    attributes among the selection will be discretized.

    :   **Parameters:**: `rangeList` - a string representing the list of attributes. Since the string will typically come from a user, attributes are indexed from 1.

---


### getAttrIndexRange

```
public java.lang.String getAttrIndexRange()
```

:   returns the attribute range(s).

    :   **Returns:**: the attribute range(s).

---


### attrIndexRangeTipText

```
public java.lang.String attrIndexRangeTipText()
```

:   Returns the tip text for this property

    :   **Returns:**: tip text for this property suitable for displaying in the explorer/experimenter gui

---


### getAttributes

```
public boolean[] getAttributes()
```

---


### getMinValue

```
public double[] getMinValue()
```

---


### getMaxValue

```
public double[] getMaxValue()
```

---


### getMeanValue

```
public double[] getMeanValue()
```

---


### getStddevValue

```
public double[] getStddevValue()
```

---


### getNumInstances

```
public int getNumInstances()
```

---


### getClusterType

```
public SelectedTag getClusterType()
```

:   Gets the cluster type.

    :   **Returns:**: the cluster type **See Also:**: `SubspaceCluster.TAGS_CLUSTERTYPE`

---


### setClusterType

```
public void setClusterType(SelectedTag value)
```

:   Sets the cluster type.

    :   **Parameters:**: `value` - the new cluster type. **See Also:**: `SubspaceCluster.TAGS_CLUSTERTYPE`

---


### clusterTypeTipText

```
public java.lang.String clusterTypeTipText()
```

:   Returns the tip text for this property

    :   **Returns:**: tip text for this property suitable for displaying in the explorer/experimenter gui

---


### getClusterSubType

```
public SelectedTag getClusterSubType()
```

:   Gets the cluster sub type.

    :   **Returns:**: the cluster sub type **See Also:**: `SubspaceCluster.TAGS_CLUSTERSUBTYPE`

---


### setClusterSubType

```
public void setClusterSubType(SelectedTag value)
```

:   Sets the cluster sub type.

    :   **Parameters:**: `value` - the new cluster sub type. **See Also:**: `SubspaceCluster.TAGS_CLUSTERSUBTYPE`

---


### clusterSubTypeTipText

```
public java.lang.String clusterSubTypeTipText()
```

:   Returns the tip text for this property

    :   **Returns:**: tip text for this property suitable for displaying in the explorer/experimenter gui

---


### isRandom

```
public boolean isRandom()
```

:   checks, whether cluster type is random

    :   **Returns:**: true if cluster type is random

---


### isUniform

```
public boolean isUniform()
```

:   checks, whether cluster type is uniform

    :   **Returns:**: true if cluster type is uniform

---


### isGaussian

```
public boolean isGaussian()
```

:   checks, whether cluster type is gaussian

    :   **Returns:**: true if cluster type is gaussian

---


### isContinuous

```
public boolean isContinuous()
```

:   checks, whether cluster sub type is continuous

    :   **Returns:**: true if cluster sub type is continuous

---


### isInteger

```
public boolean isInteger()
```

:   checks, whether cluster sub type is integer

    :   **Returns:**: true if cluster sub type is integer

---


### getMinInstNum

```
public int getMinInstNum()
```

:   Gets the lower boundary for instances per cluster.

    :   **Returns:**: the the lower boundary for instances per cluster

---


### setMinInstNum

```
public void setMinInstNum(int newMinInstNum)
```

:   Sets the lower boundary for instances per cluster.

    :   **Parameters:**: `newMinInstNum` - new lower boundary for instances per cluster

---


### minInstNumTipText

```
public java.lang.String minInstNumTipText()
```

:   Returns the tip text for this property

    :   **Returns:**: tip text for this property suitable for displaying in the explorer/experimenter gui

---


### getMaxInstNum

```
public int getMaxInstNum()
```

:   Gets the upper boundary for instances per cluster.

    :   **Returns:**: the upper boundary for instances per cluster

---


### setMaxInstNum

```
public void setMaxInstNum(int newMaxInstNum)
```

:   Sets the upper boundary for instances per cluster.

    :   **Parameters:**: `newMaxInstNum` - new upper boundary for instances per cluster

---


### maxInstNumTipText

```
public java.lang.String maxInstNumTipText()
```

:   Returns the tip text for this property

    :   **Returns:**: tip text for this property suitable for displaying in the explorer/experimenter gui

---


### setNumInstances

```
public void setNumInstances(java.util.Random r)
```

:   Sets the real number of instances for this cluster.

    :   **Parameters:**: `r` - random number generator

---


### setValuesList

```
public void setValuesList(java.lang.String fromToList)
                   throws java.lang.Exception
```

:   Sets the ranges for each attribute.

    :   **Parameters:**: `fromToList` - the string containing the upper and lower boundary for instances per cluster separated by .. **Throws:**: `java.lang.Exception` - if values are not correct in number or value

---


### getValuesList

```
public java.lang.String getValuesList()
```

:   returns the range for each attribute as string

---


### valuesListTipText

```
public java.lang.String valuesListTipText()
```

:   Returns the tip text for this property

    :   **Returns:**: tip text for this property suitable for displaying in the explorer/experimenter gui

---


### setMeanStddev

```
public void setMeanStddev(java.lang.String meanstddev)
                   throws java.lang.Exception
```

:   Sets mean and standarddeviation.

    :   **Parameters:**: `meanstddev` - the string containing the upper and lower boundary for instances per cluster separated by .. **Throws:**: `java.lang.Exception` - if values are not correct in number or value

---


### getMeanStddev

```
public java.lang.String getMeanStddev()
```

:   returns the current mean/stddev setup

---


### meanStddevTipText

```
public java.lang.String meanStddevTipText()
```

:   Returns the tip text for this property

    :   **Returns:**: tip text for this property suitable for displaying in the explorer/experimenter gui

---


### setValuesList

```
public void setValuesList(java.lang.String fromToList,
                          double[] first,
                          double[] second,
                          java.lang.String optionLetter)
                   throws java.lang.Exception
```

:   Sets the ranges for each attribute.

    :   **Parameters:**: `fromToList` - the string containing the upper and lower boundary for instances per cluster separated by ..: `first` - the "from's": `second` - the "to's": `optionLetter` - the option, from which the list came **Throws:**: `java.lang.Exception` - if values are not correct in number or value

---


### getRevision

```
public java.lang.String getRevision()
```

:   Returns the revision string.

    :   **Returns:**: the revision


---


|  |  |  |
| --- | --- | --- |
| **Overview** \| **Package** \| **Class** \| **Tree** \| **Deprecated** \| **Index** \| **Help** \| **Weka's home** | |  |
| **PREV CLASS**   NEXT CLASS | **FRAMES**    **NO FRAMES**     **All Classes** |  |
| SUMMARY: NESTED \| FIELD \| CONSTR \| METHOD | DETAIL: FIELD \| CONSTR \| METHOD |  |


---
